# Supplementary material for: A Case-Control Study on Factors of HPV Vaccination for Mother and Daughter in China
Source: Vaccines (Basel). 2023 May 12;11(5):976. doi: 10.3390/vaccines11050976 (PMC10224422; doi:10.3390/vaccines11050976)
Supplement: Supplementary file 1 [file vaccines-11-00976-s001.zip › vaccines-2344687-supplementary.pdf]

**Supplementary Materials:**

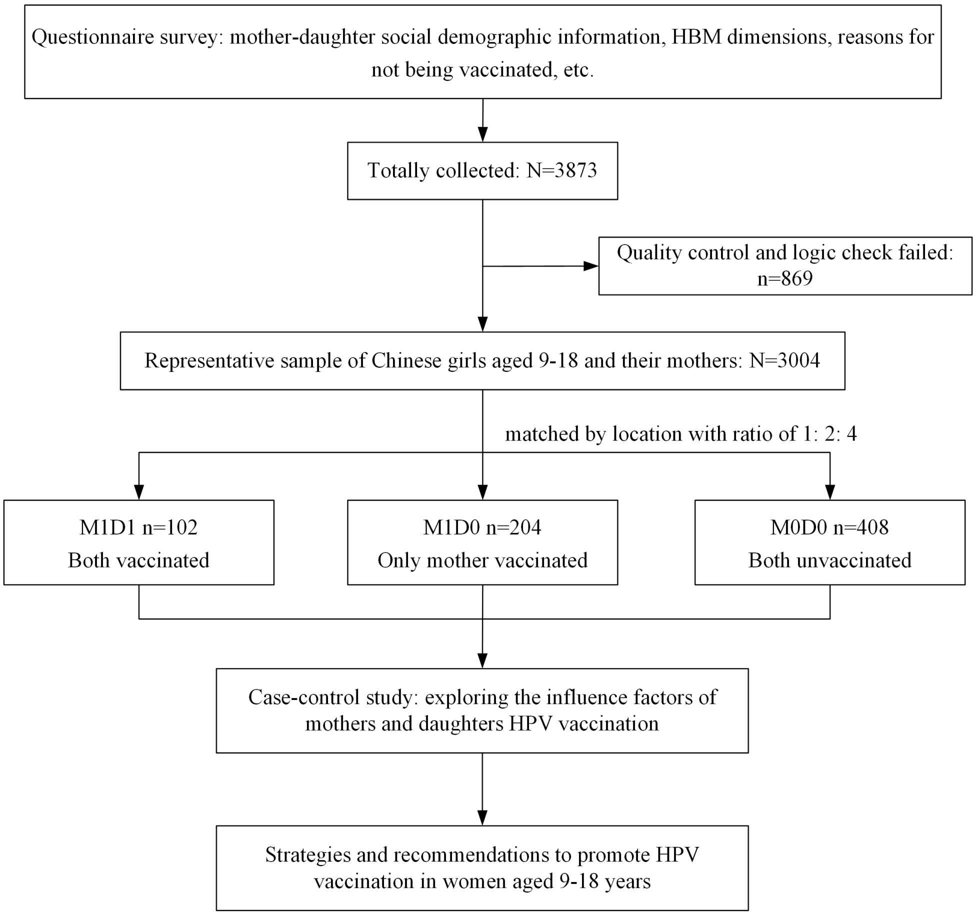


**Figure S1.** Research path map and sample collection status.

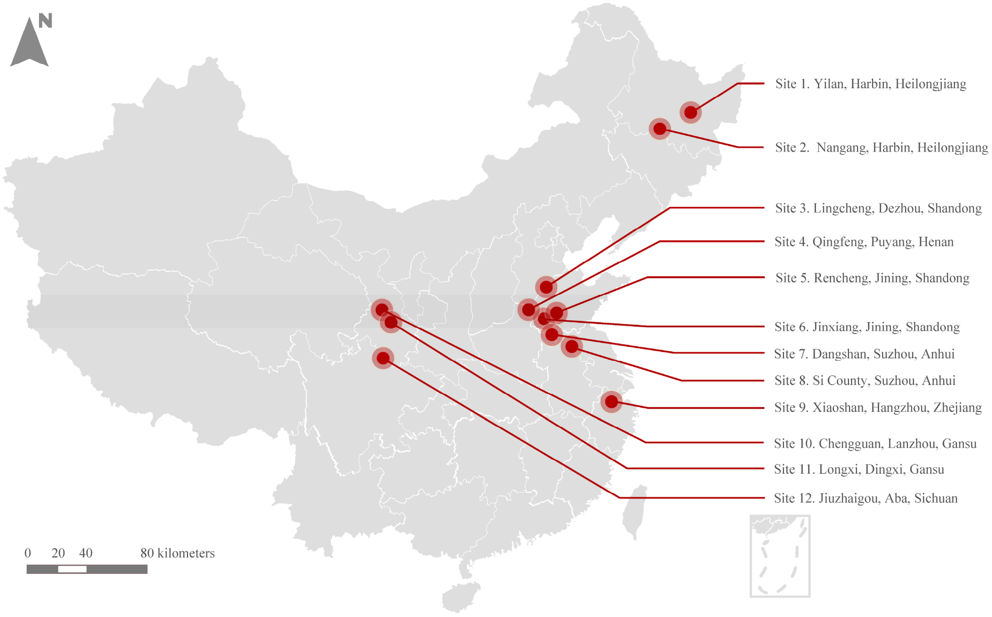


**Figure S2.** Distribution of investigation sites.

**Table S1.** Questions and scoring of each dimension of health belief model.

| Dimension | Number | Question | Score/Points |
|---|---|---|---|
| A. HPV and HPV vaccine knowledge | A1 | Most cervical cancer is caused by a human papillomavirus infection | 1: "Yes"; 0: "No", "Not sure" |
| | A2 | The population infected with human papillomavirus HPV is | 1: "Both male and female"; 0: "Male only", "Female only", "Not sure" |
| | A3 | The main route of transmission of HPV is | 1: "sexual transmission"; 0: "mother to child transmission", "Close contact transmission", "Indirect contact transmission" |
| | A4 | The body can automatically clear the HPV after being infected | 1: "Yes"; 0: "No", "Not sure" |
| | A5 | Is it certain to get sick after an HPV infection | 1: "No"; 0: "Yes", "Not sure" |
| | A6 | How many doses of the 9-valent HPV vaccine need to be administered | 1: "3 times"; 0: "1 time", "2 times", "4 times", "Not sure" |
| | A7 | Who needs HPV vaccination | 1: "Both male and female"; 0: "Male only", "Female only", "Not required for either sex" |
| | A8 | The best time to get HPV vaccinated is | 1: "Before the first sexual behavior"; 0: "After birth", "After the first sexual behavior ", "Any time", "Not sure" |
| B. Perception of behavioral benefit | B1 | I think HPV vaccines are important for protecting myself from HPV infection and related diseases | 4-0: "Strongly agree"–"Strongly disagree" |
| | B2 | I think the safety of the HPV vaccine can be guaranteed | 4-0: "Strongly agree"–"Strongly disagree" |
| | B3 | I think the HPV vaccine is effective | 4-0: "Strongly agree"–"Strongly disagree" |
| C. Perception of barriers | C1 | Compared with myself, I treat my daughter to take medicine or play HPV vaccine more cautiously | 4-0: "Strongly agree"–"Strongly disagree" |
| | C2 | The current domestic bivalent HPV vaccine price is about 300 CNY. Do you think the price is reasonable? | 3: "Reasonable"; 2: "Acceptable"; 1:"A little expensive"; 0: "Very expensive" |
| D. Perception of disease susceptibility | D1 | As long as I keep a normal lifestyle, I will not be infected by HPV | 4: "No"; 0: "Yes", "Not sure" |
| | D2 | I think infection by HPV is because of sexual arbitrariness | 4-0: "Strongly agree"–"Strongly disagree" |
| | D3 | My daughter is in good health and not afraid of an HPV infection | 4-0: "Strongly agree"–"Strongly disagree" |
| E. Perception of disease severity | E1 | HPV infection has serious consequences | 1: "Yes"; 0: "No", "Not sure" |
| | E2 | HPV infection can bring serious harm to my daughter's health | 4-0: "Strongly agree"–"Strongly disagree" |
| | E3 | I think cervical cancer is a serious disease | 4-0: "Strongly agree"–"Strongly disagree" |
| | E4 | I worry that HPV infection will affect the child' s fertility | 4-0: "Strongly agree"–"Strongly disagree" |
| F. Trust in formal information | F1 | I trust the HPV vaccine knowledge and services provided by vaccination clinic and doctor | 4-0: "Strongly agree"–"Strongly disagree" |
| | F2 | I trust the HPV vaccine knowledge and services provided by CDC, hospitals and other professional institutions | 4-0: "Strongly agree"–"Strongly disagree" |
| | F3 | I trust HPV vaccine manufacturers and companies | 4-0: "Strongly agree"–"Strongly disagree" |

| | | |
|---|---|---|
| F4 | I trust the HPV vaccine information provided by the government | 4-0: "Strongly agree"–"Strongly disagree" |

**Table S2.** Classification of reasons for not getting the HPV vaccine.

| Category | Reasons for mothers | Reasons for daughters |
|---|---|---|
| Non-vaccine hesitancy | (1) Older ages<br>(2) Illness or medical reasons<br>(3) HPV vaccines shortage | (1) Daughter's illness or medical reasons<br>(2) HPV vaccines shortage |
| Vaccine/vaccination-specific issues | (1) Doubts about the vaccine efficacy<br>(2) Doubts about the vaccine safety<br>(3) Expensiveness<br>(4) Troublesome vaccination procedure | (1) Doubts about the vaccine efficacy.<br>(2) Doubts about the vaccine safety<br>(3) Expensiveness<br>(4) Troublesome vaccination procedure |
| Individual and group influences | (1) Don't know where to get vaccinated<br>(2) Infection with HPV usually has no serious consequences<br>(3) Never heard of the HPV vaccine<br>(4) No time to vaccinate<br>(5) My risk of HPV infection is low<br>(6) Be afraid of pain<br>(7) Poor service and attitude at the vaccination sites | (1) Don't know if my daughter can be vaccinated<br>(2) Wait for my daughter growing up and make the decision herself.<br>(3) My daughter has not started sexual behavior<br>(4) Wait for my daughter being old enough to receive the 9-valent vaccine<br>(5) Don't know where to get vaccinated<br>(6) Worry my daughter will have high-risk sexual behavior after vaccination<br>(7) Never heard of the HPV vaccine<br>(8) Infection with HPV usually has no serious consequences<br>(9) No time to vaccinate<br>(10) My daughter's risk of HPV infection is low<br>(11) My daughter is afraid of pain<br>(12) Poor service and attitude at the vaccination sites<br>(13) My daughter is too young |
| Contextual influences | (1) Less people around me were vaccinated or they do not recommend this vaccine<br>(2) The HPV vaccine is not yet widely available<br>(3) Not convenient to reach the vaccination site | (1) Less people around me were vaccinated or they do not recommend this vaccine<br>(2) The HPV vaccine is not yet widely available<br>(3) Not convenient to reach the vaccination site |
